# Mammary-like adenocarcinoma of the vulva associated to Paget's disease: a case report

**DOI:** 10.11604/pamj.2014.19.188.4521

**Published:** 2014-10-23

**Authors:** Sawsen Meddeb, Mohamed Salah Rhim, Sarra Mestiri, Mouna Kouira, Mohamed Bibi, Hedi Khairi, Mohamed Tahar Yacoubi

**Affiliations:** 1 Department of Gynecology and obstetrics, Farhat Hached University Hospital, Sousse, Tunisia; 2 Research laboratory in quality of Maternal Health Care Tunisia; 3 Department of Pathology, Farhat Hached University Hospital, Sousse, Tunisia

**Keywords:** Mammary-like adenocarcinoma, vulva, Paget′s disease

## Abstract

Mammary-like adenocarcinoma of the vulva associated to Paget's disease is exceedingly rare. So, it is very important to perform all the pathological and immunohistochemical investigations to achieve differential diagnosis from both a metastatic lesion from an orthotopic breast cancer and a vulvar adnexal tumor. This report describes a case of vulvar Paget's disease associated with underlying mammary-like adenocarcinoma diagnosed in the Department of Obstetrics and Gynecology of Farhat Hached university hospital of Sousse in Tunisia. We also review previously reported cases of primary breast-like carcinoma of the vulva with or without Paget's disease.

## Introduction

Primary adenocarcinomas of the vulva are rare. However, a rare form of adenocarcinoma can arise from mammary-like vulvar glands displaying features of both eccrine and mammary glands. We report a case of vulvar Paget's disease associated with underlying mammary-like adenocarcinoma. To explore the possibility that this was a case of primary breast carcinoma of the vulva, we investigated the immunohistochemical characteristics of the tumor cells. We also review previously reported cases of primary breast-like carcinoma of the vulva with or without Paget's disease.

## Patient and observation

**Figure 1:**
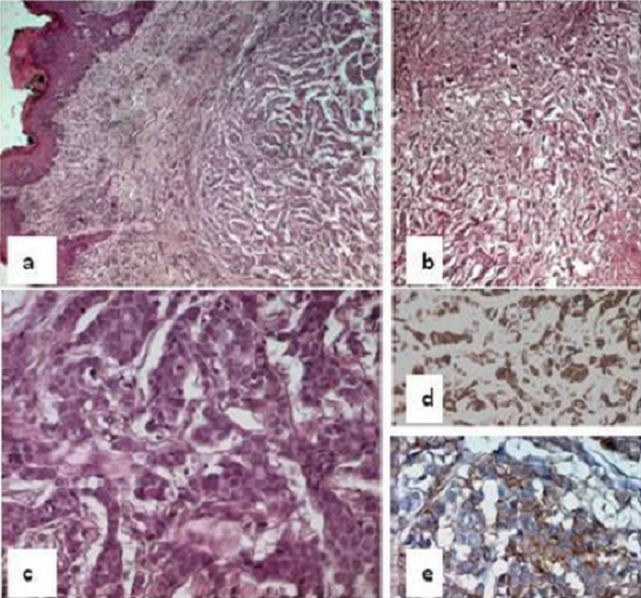
Histologic features of the tumor: (a) HE Staining x 40: modular malignant epithelial proliferation overlayed by hyperplastic epidermis with paget's lesion. (b): HE Staining x 40: trabecular proliferation centrated by necrotic areas. (c): HE Staining x 400: Cuboïd epithelial cells with indistinct borders amphophilic cytoplasm and hyperchromatic nuclei. Figures of mitoses are frequent. (d): positive immunostain of this tumor cells by the anti cytokeratine 7. (e): Diffuse and intensive immunostain of the tumor cells by the anti Her-2/neu

A 41-year-old woman, gravida 4 para 4, presented with a left vulvar labia mass. There was no previous history of malignancy or breast disease. Her family history was not remarkable for carcinomas. Physical examination found a 2 cm in diameter, erythematous and ulcerative nodule located between the left labium major and labium minor. The clitoris, right labia, vestibule and vaginal wall were intact. An enlarged fixed lymph node was palpated in each groin. An excisional biopsy specimen of the lesion was performed. Histopathological examination found a neoplastic proliferation of epithelial cells, arranged in cords, trabeculae and nests. The neoplastic cells were cuboid or round-shaped with amphophilic cytoplasm. The nuclei were enlarged, slightly pleomorphic with coarse chromatin. Nucleoli were small or absent. Glandular structures were rarely seen. Mitotic count found at least 10 mitoses/ 10 HPF. A focal area of coagulative necrosis was seen. The stroma was made of delicate strands of fibrous tissue. The overlying squamous epithelium was partially replaced by nests of large neoplastic cells, located within the basal and parabasal layers. These cells had an abundant clear or amphophilic cytoplasm. Their nuclei are large, vesicular or having a dense chromatin. One or more enlarged nucleoli are present. Some mitotic figures are seen (Figure 1: a, b, c). Immunohistochemical study showed that the tumor cells were positive for low-weight cytokeratin (CK7), EMA and Her-2/neu (Figure 1: d, e). It was negative for high-weight cytokeratin (CK20), melanocytic markers (HMB 45, Melan A) and both hormonal receptors. The immunostaining results for Her-2 expression were scored 2+. Chromogenic in situ hybridization (CISH) with monoprobe for Her-2 amplification was negative. The final diagnosis proposed was a Mammary gland-like adenocarcinoma of the vulva with an overlying Paget's disease. The breast examination was unremarkable. No metastasis was seen on the chest X-ray or abdominal ultrasound. The patient underwent a radical vulvectomy with bilateral inguinal lymph nodes dissection. The tumor measured 1.5 cm in great diameter showing the similar histopathological features seen on the biopsy specimen, described previously with numerous images of lymphatic invasion. It was confined to the vulva. Surgical margins were free of tumor. Four metastatic and bilateral regional nodes were found within the 13 sampled. The tumor was staged T1N2bM0 (TNM, 7^th^edition) and IIIB (FIGO 2009). The patient had simple operating consequences. She's, at present, under radiotherapy and then proposed for adjuvant chemotherapy.

## Discussion

Vulva accounts for the majority of extra-mammary Paget's disease location (76%) [1]. The specific occurrence of primary breast-like carcinoma of the vulva is exceedingly rare with approximately 17 cases, including the present case, being reported in the literature between 1872 and 2013 [2]. n approximately 10-20% of cases, vulvar Paget's disease is known to be associated with invasive adenocarcinoma [3]. Involvement of the overlying epidermis with Paget's disease was observed in seven cases, including the present case.

A review of the literature found that the mean age of the patients was 60 year old. The most common symptoms are pruritus, burning pain, lump and occasionally painful erosion [3]. A visible lesion, typically an erythematous plaque is present in almost all patients. The average dimension of the lesion is of about 2.5cm and the lesion is in the most of cases unilateral (bilateral in one case). Major labia are the most often involved site (15cases), followed by minor labia (5 cases) and clitoris (2 cases) [2]. Initially, these tumors were presumed to arise from ectopic breast tissue believed to exist in the vulvar region. Moreover, mammary-like glands, originally termed anogenital sweat glands, share many features common to both eccrine and mammary glands [4]. These glands differ from normal sweat glands by demonstrating positivity for hormone receptors (estrogen and progesterone). Additionally, these glands are also ultrastructurally distinct from both sweat and mammary glands [4].

Differential diagnosis includes adenocarcinoma arising from Bartholin glands, sweat gland carcinoma, and adenocarcinomas of metastatic origin [3]. Distinguishing between these tumors and adenocarcinoma of mammary-like glands is particularly difficult in the absence of normal mammary-like glands and transition zones that exist between benign and tumorous areas. In the present case, we have found in the vicinity of the tumor some glandular structures of eccrine type, lined by cuboid cells similar to those seen in the tumor. Furthermore, the following criteria should be sufficient to categorize breast carcinoma of primary vulvar origin [4]: A morphologic pattern consistent with breast carcinoma; the expression of Estrogen Receptor and/or Progesterone Receptor and positivity for common breast cancer markers such as EMA, CEA and glandular keratins. The present case fulfilled two of the above criteria, and therefore it is reasonable to diagnose the present vulvar tumor as breast-like carcinoma.

Vulvar Paget's disease has recently been classified into three distinct types based on the origin of the neoplastic cells [5]: Primary vulvar intraepithelial Paget's disease (type 1a), primary vulvar intraepithelial Paget's disease with invasion (type 1b), and vulvar Paget's disease presenting as a manifestation of a primary underlying adenocarcinoma of the vulva (type 1c). The distinction between these 3 types is essential to avoid potential confusion and unnecessary surgery [5]. Besides, the Yale experience reveals an association between outcome of extra-mammary Paget's disease and Her-2/neu over expression. It was found in this work that Her-2/neu over expression rate was higher in patients with invasive disease (71% versus 54%) [5]. This finding supports the possible therapeutic use of anti-Her-2/neu antibodies (Trastuzumab). Besides, the Ki-67 and Cyclin D1 expression are found to be expressed at higher levels in invasive lesions than in situ lesions [6].

The majority of case reports in the literature detail the surgical management of this tumor involving a wide local excision or radical vulvectomy with node dissection followed by adjuvant chemotherapy, hormonotherapy or radiation, similar to the management of an isolated malignant breast mass. In 10-20% of cases, vulvar Paget's disease is associated to other malignancies at other sites, as the breast, the rectum, the genitourinary tract and the cervix [7]. A Spanish study reports that vulvar extramammary Paget's disease was associated with adnexal adenocarcinoma in 4% of cases and with a distant malignancy in 20% of cases [7]. Due to the high incidence of coexisting malignancies at other sites, diagnosis of vulvar Paget's disease should lead to further investigations such as mammography, colonoscopy, colposcopy and cervical cytology [8]. We also suggest that patients with ectopic breast cancer should be followed-up as though they had à breast cancer, with à chest X-ray, liver ultrasound examination and total body bone scintigraphy to assess the most probable metastatic sites of the lesion. The evaluation of serum level of CA15.3 also appears to main to be a reliable biological marker to evaluate relapses and responses to chemotherapy in these patients [8].

Abbott and Ahmed [9] concluded that aggressive surgical therapeutic regimens, particularly in the case of tumors localized to the skin, should be reassessed, given the morbidity faced by such therapy. They rather suggest that Mohs micrographic surgery can be adopted for tumors localized to the skin, especially in elderly patients or those with severe comorbid conditions. Given the high frequency of nodal disease at presentation with adenocarcinoma of mammary-like glands, the role of sentinel lymphadenectomy must be explored, especially when no evidence of such disease is apparent either clinically or radiographically [10].

## Conclusion

When Paget's disease with underlying mammary-like adenocarcinoma of the vulva is established, it is very important to perform all the pathological and immunohistochemical investigations to achieve differential diagnosis from both a metastatic lesion from an orthotopic breast cancer and a vulvar adnexal tumor. Treatment and follow-up should be the same as if it was an orthotopic breast neoplasm.
